# Contribution of the Nelson R. Mandela School of Medicine to a socially accountable health workforce

**DOI:** 10.4102/phcfm.v11i1.1962

**Published:** 2019-04-23

**Authors:** Amy Clithero-Eridon, Danielle Albright, Cameron Crandall, Andrew Ross

**Affiliations:** 1 Department of Family and Community Medicine, University of New Mexico, Albuquerque, New Mexico, United States; 2 Department of Emergency Medicine, University of New Mexico, Albuquerque, New Mexico, United States; 3 Department of Family Medicine, University of KwaZulu-Natal, Westville, South Africa

**Keywords:** physician workforce, social accountability, physician recruitment, physician retention, district hospitals

## Abstract

**Background:**

A socially accountable health professional education curriculum aims to produce fit-for-purpose graduates to work in areas of need. ‘Fit-for-purpose’ can be assessed by monitoring graduate practice attributes.

**Aim:**

The aim of this article was to identify whether graduates of ‘fit-for-purpose’ programmes are socially accountable.

**Setting:**

The setting for this project was all 37 district hospitals in the KwaZulu-Natal province in Durban, South Africa.

**Methods:**

We surveyed healthcare professionals working at district hospitals in the KwaZulu-Natal province. We compared four social accountability indicators identified by the Training for Health Network Framework, comparing medical doctors educated at the Nelson R. Mandela School of Medicine (NRMSM) with medical doctors educated at other South African and non-South African medical schools. In addition, we explored medical doctors’ characteristics and reasons for leaving or staying at district hospitals.

**Results:**

The pursuit of specialisation or skills development were identified as reasons for leaving in the next 5 years. Although one-third of all medical doctors reported an intention to stay, graduates from non-South African schools remained working at a district hospital longer than graduates of NRMSM or other South African schools and they held a majority of leadership positions. Across all schools, graduates who worked at the district hospital longer than 5 years cited remaining close to family and enjoyment of the work and lifestyle as motivating factors.

**Conclusion:**

Using a social accountability approach, this research assists in identifying areas of improvement in workforce development. Tracking what medical doctors do and where they work after graduation is important to ensure that medical schools are meeting their social accountability mandate to meet community needs.

## Background

The World Health Organization (WHO) recognises significant global challenges in recruiting and retaining medical doctors for rural and remote locations.^1^ In response to these challenges, the WHO Global Strategy for Human Resources for Health 2030 tasked institutions of higher learning (IHL) with training increasing numbers of healthcare professionals (HCPs) as a key component of health equity.^2^ However, simply producing more HCPs is not sufficient to improve access as inequitable distribution of HCPs contributes to health inequities within and between populations. The inequitable distribution of health services and HCPs within South Africa (SA) exemplifies this challenge,^3^ with many rural areas experiencing greater shortages of staff than urban areas^4,5^ and only 12% of doctors choosing to work in rural areas, even though 44% of the population resides rurally.^6,7^ An additional challenge in SA is that 80% of doctors prefer to work in the private sector, resulting in gaps in the available workforce in public health care even though 85% of the South African population relies on an under-resourced public health sector for health care.^8^ To address these challenges, it is important to understand doctors’ motivations for remaining in areas of high need and their reasons for leaving.^9,10,11^

There have been a number of initiatives to address the staffing challenges in rural and underserved areas in SA, such as loan repayment options that have had an impact on the staffing of rural facilities.^12,13^ Another financial incentive is a rural allowance for medical doctors to work in underserved areas. In 1998, to address the shortages of healthcare workers in areas of need, particularly rural areas, the South African government through the Department of Health introduced a 1-year community service obligation for South African medical doctors. This has been described as having ‘the strongest impact on countering health worker shortages and on the maldistribution of health professionals in South Africa’.^14^ However, although compulsory community service for new graduates to work in underserved settings has positively affected retention, there is evidence to suggest that provision of professional support and development is essential if doctors are to remain for longer than the compulsory time period.^13,15^

International experience from the Training for Health Equity Network (THEnet), an international collaboration of 13 health professional schools pursuing social accountability mandates,^16^ suggests that strategies to recruit and retain a rural workforce might be even more effective if health professional education curricula at local training institutions adhered to a social accountability framework to produce fit-for-purpose graduates. The WHO definition of social accountability is as follows:

The obligation to orient education, research, and service activities towards priority health concerns of the local community, the region and/or nation (schools) one has a mandate to serve. These priorities are jointly defined by government, health service organizations, and the public.^17^

Fit-for-purpose graduates are trained technically, socially and culturally to meet the health and social needs of the communities they serve. One method ‘fit-for-purpose’ can be assessed is by monitoring the location where graduates practice. We selected HCPs working at district hospitals within the KwaZulu-Natal (KZN) province for this study. District hospitals deliver basic health services to a largely economically disadvantaged and marginalised population in developing countries. In fact:

The district health system (DHS) is often seen as the means of achieving an equitable, efficient and effective health system based on the principles of the Primary Health Care (PHC) approach. This is because a main strength of the health district model is the combination of strong values – equity, efficacy, efficiency, autonomy and solidarity with conceptual neatness and operational relevance.^18^

Staffing of DHS is crucial for health service delivery within a well-functioning equitable healthcare system.

The Nelson R. Mandela School of Medicine (NRMSM) is part of the University of KwaZulu-Natal (UKZN) in Durban, SA. It is one of SA’s nine health professional schools and is based in the province of KZN, the second largest province in SA.^19,20^ The NRMSM medical school graduates 200 medical students each year, following successful completion of a 6-year programme. Although the mission statement of NRMSM commits the institution to ‘function holistically, ethically and in a socially responsive manner within the African health care environment’,^21^ most of the curriculum is focused on understanding and managing disease. An exception to this biomedical focus is the training delivered to all medical students throughout the curriculum by the Department of Public Health Medicine and Family Medicine at NRMSM. The department is responsible for a number of modules that emphasise caring for the underserved and understanding ‘upstream’ factors which contribute to ill health and community-orientated primary care (Box 1). While there are many factors that impact retention of healthcare workers in rural areas,^22^ the potential influence of the overall NRMSM curriculum on why NRMSM graduates choose to work in the public sector and whether they remain beyond their obligations is not well understood. To facilitate introspection, this study selected four indicators from THEnet aspirational framework to better understand how NRMSM is progressing towards social accountability.^23^

BOX 1 Nelson R. Mandela School of Medicine socially accountable curriculum.

*Making a difference* is part of becoming a professional module where students learn to Care for the Underserved. Students are required to complete 16 h of community service in a disadvantaged community and explore issues with the participants around the meaning of wellness and disease.

*Selective placements (years 2–4):* Students are introduced to a population perspective via linkage of disease to community and social determinants of health. Students must engage in a longitudinal 3-year immersion experience which occurs in a community-based primary care clinic close to their home and in their own community as part of the selective programme. Using the community as a classroom, students ‘diagnose’ a community issue, research the issue and then ‘treat’ the community by implementing an evidence-based intervention. This capstone project is done in collaboration with community members, who assist in identifying a community health priority. In 2015, a study showed that student placement during their selections reflected their home community; specifically, 37% of students came from rural communities and 39% chose a rural hospital selective.^24^

*Family medicine:* Family medicine (FM) is housed within the School of Nursing and Public Health at UKZN and emphasises social accountability in curriculum planning. As the unifying component of the curriculum and with a presence in every year, it has a prominent role of drawing together the important aspects of the curriculum (see Table 1). In their fourth year, FM students are placed in PHC clinics, and in the fifth year they are based at an urban district hospital. In their final year, students are placed in rural district hospitals to gain experience with common community illnesses, with a particular emphasis on the human immunodeficiency virus (HIV), tuberculosis, working with non-governmental organisations and making appropriate referrals to tertiary care.

**TABLE 1:** Family medicine within a comprehensive 6-year medical school curriculum.

Medical school year	Topic	Delivery
MS Year 1	HIV and me	5 lectures or workshops
	Self-care	5 lectures
	Family systems and family systems assessment	4 lectures
	Addictive disorders	1 lecture
	Making a difference module	16 h of facilitation of community service in an area of need
MS Year 2	Disease prevention and health promotion	4 lectures
	Selective 1	160 notional hours – community-based programme
MS Year 3	Selective 2	160 notional hours – community-based programme
	Disease prevention and health promotion	4 lectures
MS Year 4	Selective 3	160 notional hours – community-based programme
	Primary health care	6 weeks module
MS Year 5	Primary health care	6 weeks module
MS Year 6	Primary health care	7 weeks at a rural district hospital

HIV, human immunodeficiency virus; MS, medical school.

A detailed description of the primary care and public health curricular components is presented elsewhere.^24^

The aim of this article was to compare the ‘fit-for-purpose’ attributes of those educated at the NRMSM with those educated at other institutions using select social accountability indicators from THEnet framework. It also aimed to identify the characteristics and perspectives of doctors who chose to stay in or leave public service, specifically at district hospitals, which care for underserved populations.

## Methods

In this study, we analysed secondary data. The primary data were collected as part of a broader project on health professional staffing at district hospitals in KZN province. Briefly, Ross et al.^25^ conducted a descriptive cross-sectional study at all 37 district hospitals in KZN to assess the extent to which NRMSM contributes to the staffing of doctors, physiotherapists, pharmacists, dietitians and dental therapists working at district hospitals in KZN. A piloted questionnaire was used to gather information on where the HCPs graduated from, how long they had been working at the district hospitals, what their current positions in the hospital were as well as their short-term and long-term plans. Data were obtained from 514 HCPs at 29 out of 37 (78%) district hospitals. Based on data returned from the human resource departments at these hospitals, this represents 56% of eligible HCPs working in these hospitals. This article will focus on medical doctor responses (*N* = 231).

### Indicators

Four indicators from the THEnet Social Accountability Framework^23^ that describe postgraduate practice were selected and responses of NRMSM medical school graduates were compared with those of medical graduates from other South African and non-South African health professional schools, all of whom were practicing in district hospitals. The indicators are the number and proportion of graduates who (1) mirror the population they serve, (2) remain working with assigned communities 5 years after graduation, (3) pursue further specialisation and (4) are in leadership roles. We further assessed the intentions and characteristics of medical doctors who stated that they plan to leave their assigned community after completing their service obligation (community service and any work back or pay back obligation).

For descriptive purposes, we grouped the responses of medical doctors according to the schools from which they were qualified and assigned them into three categories: NRMSM, South African schools excluding NRMSM and other schools outside of SA.

We asked the participants to identify and rank as primary, secondary or tertiary their reasons for staying at a district hospital. They chose from five options commonly identified in the literature as reasons for staying for a long term in a position.^12,26^ The participants were also given the option to provide free text in an open-ended ‘other’ category. We combined text responses into broad categories if appropriate; otherwise, they were categorised as an ‘other’ response. We reviewed only primary responses as secondary and tertiary responses were not frequently selected.

One of THEnet indicators for social accountability of graduates is the number and proportion of graduates in healthcare leadership roles. In SA, there are six titles given to medical doctors. We defined participants based on the following positions: chief medical officer, medical manager, consultant and clinical manager as holding leadership roles.

### Statistical analysis

Data generated from the original survey included closed- and open-ended questions. Researchers at NRMSM entered the survey data into a Microsoft Excel (2016) spreadsheet and provided a copy to investigators at the University of New Mexico, School of Medicine in Albuquerque, NM, United States (US).^27^ We imported these data into REDCap, a database management system. REDCap encrypts and stores data on secure servers with a firewall restricting unauthorised access.^28^ We imported and analysed the data using SAS 9.4 statistical software.^29^ To compare continuous variables (e.g. age) between groups, we used Wilcoxon rank sum methods. To compare categorical variables between groups, we used the chi-square test or Fisher’s exact test (FET) where appropriate.

### Ethical considerations

The Biomedical Research and Ethics Committee of the University of KwaZulu-Natal approved the study design (BE 330/16). Each participant provided written informed consent prior to participation in the study.

## Results

### Sample demographic characteristics

Responses were received from 231 medical doctors (representing a 45% response rate), including 89 (38%) NRMSM graduates, 64 (28%) other South African medical schools and 78 (34%) from non-South African medical schools. Graduates represented seven South African schools (non-NRMSM), five schools on the African continent and 10 schools from non-African countries. Slightly less than half (46%) of the participants were identified as females. The median age of the participants was 31 years, with a range of 24–67 years. Outliers were those aged 55 years and above (*N* = 14). Male participants were significantly older than female participants (the male participants’ median age was 35 years and the female participants’ median age was 29 years, *p* < 0.0001). The age of NRMSM students was similar to the other graduates (*p* < 0.0001). The majority of participants were identified as black people (56%), followed by white people (26%), Indians (15%) and mixed race people (2%). (see Table 2)

**TABLE 2:** Sample demographic characteristics for the total sample and by medical school qualification.

Variable	Total		Nelson R. Mandela School of Medicine		Other South African schools^†^		Non-South African schools^‡^		KwaZulu-Natal province demographics^30^	
	*N*	%	*N*	%	*N*	%	*N*	%	*N*	%
**Gender (missing, *N* = 2)**										
Female	106	46	47	44	28	26	31	29	5 758 945	52
Male	123	53	41	33	35	28	47	38	5 306 295	48
**Race (missing, *N* = 18)**										
Black	120	56	64	53	27	23	29	24	9 625 934	87
White	56	26	1	2	28	50	27	48	432 056	0.04
Indian	32	15	17	53	9	28	6	19	873 161	0.09
Mixed race	5	2	1	20	0	0	4	80	134 089	0.01
**Total**	**231**	**100**	**89**	**38**	**64**	**28**	**78**	**34**	**11 065 240**	**19.9**

† , University of Cape Town Medical School (Cape Town), University of the Free State (Free State), Sefako Makgatho Health Sciences University (Pretoria), University of Pretoria (Pretoria), Stellenbosch University (Cape Town), Walter Sisulu University (Mthatha), University of the Witwatersrand (Pretoria).

‡ , Democratic Republic of the Congo (university specific name not identified), Makere University School of Medicine (Kampala, Uganda), University of Lagos (Lagos, Nigeria), Medical School of Tunis (Tunis, Tunisia) and the University of Zimbabwe (Harare, Zimbabwe).

### Intention to stay and bursary obligation

Only one-third (33%) of all medical doctors reported an intention to stay (*N* = 68). For NRMSM graduates, the percentage was similar (35%, *N* = 24, *p* < 0.3320). About one-third of all medical doctors (*N* = 86, 38%) reported receiving a bursary obligation. Bursary obligation varied by school, with the majority (63%) held by NRMSM graduates, 23% by other South African schools and 14% by non-South African schools (*p* < 0.0001). Of those reporting a bursary obligation, 86% (*N* = 74) answered questions regarding where they qualified from and whether they intended to leave their post within the next 5 years. About one-quarter (26%) of respondents with a bursary obligation reported an intention to stay after 5 years. Intention to stay did not vary across the schools (NRMSM, 30%; other South African schools, 28%; non-South African schools, 39%) (*p* = 0.351). (see Table 3)

**TABLE 3:** Social accountability practice characteristics for the total sample and by medical school qualification.

Length of time working at district hospital§	Total		Nelson R. Mandela School of Medicine		Other South African schools†		Non-South African schools‡	
	*N*	%	*N*	%	*N*	%	*N*	%
Less than 5 years	170	74	67	39	46	27	57	34
More than 5 years	33	14	8	24	11	33	14	42
Bursary obligation	86	38	54	63	20	23	12	14
Intention to stay¶	68	29	24	36	16	24	28	41

† , University of Cape Town Medical School (Cape Town), University of the Free State (Free State), Sefako Makgatho Health Sciences University (Medunsa), University of Pretoria (Pretoria), Stellenbosch University (Cape Town), Walter Sisulu University (Mthatha), University of the Witwatersrand (Pretoria).

‡ , Democratic Republic of the Congo (university specific name not identified), Makere University School of Medicine (Kampala, Uganda), University of Lagos (Lagos, Nigeria), Medical School of Tunis (Tunis, Tunisia) and the University of Zimbabwe (Harare, Zimbabwe).

§ , missing, *N* = 28.

¶ , missing, *N* = 24.

### Who stays and why?

Fulfilling a community service obligation was the most commonly cited reason for working at a district hospital (*N* = 70, 33%), followed by enjoyment of work and lifestyle (*N* = 58, 27%) and being close to family (*N* = 31, 15%). We observed variation in the ranking by school. A formal payback commitment in the form of a bursary obligation or community service requirement was the primary reason for both NRMSM and other South African graduates, indicating that they would remain at the district hospital, while enjoyment of the work and lifestyle was the primary driver for students from non-South African schools. We were also curious about a subset of respondents who have stayed in district hospitals for more than 5 years. Remaining close to family (33%) and enjoyment of the work and lifestyle (33%) were the most commonly cited reasons for staying for both male and female students across all medical schools.

Among all the respondents, there were no clear patterns between age, gender and intention to stay (*p* = 0.0001 FET [Fisher’s exact test]). A small number of female respondents (*N* = 5), but no men, identified family as a reason for leaving. Female respondents cited either a community service (*n* = 38, 54%) or a bursary commitment (*N* = 12, 57%) as reasons for staying, while men cited enjoyment of the work and lifestyle (*N* = 41, 72%) and being close to family (*N* = 32, 68%) as the main retention reasons.

We also looked at race as a potential factor for staying. There were 64 respondents who answered about where they qualified from, their race and stated that they did not intend to leave their post. Slightly more than half (*N* = 33, 51%) were black people, followed by white people (*N* = 18, 28%), Indians (*N* = 10, 16%) and mixed race people (*N* = 3, 5%). With regard to those qualified from NRMSM, results varied slightly, with 65% (*N* = 13) of black people intending to stay, followed by Indians (*N* = 7, 30%) and mixed race people (*N* = 1, 4%). No white people stated that they intended to stay.

For all schools, the pursuit of specialisation or skills development was the main reason for leaving within the next 5 years (*N* = 66, 47%). Nelson R. Mandela School of Medicine graduates, intending to leave their current position, were consistent with graduates from other schools, with 44% indicating specialisation and skills development as the primary reason for intention to leave (*p* = 0.0001).

### Leadership roles

A small proportion of respondents (16%, *N* = 33) held leadership roles within the district hospitals. Nearly half (48%) of the leaders qualified from non-South African schools, 27% from NRMSM and 24% from other South African schools (*p* = 0.1598).

A greater proportion of respondents with more than 5 years of experience are in leadership positions (38%) as compared to those with less experience (11%) (*p* = 0.0005). Leaders with more than 5 years of experience did not vary by school.

## Discussion

In this article, we compared ‘fit-for-purpose’ attributes of those educated at the NRMSM and who are receiving incentives for working in underserved communities with those educated at other institutions. While medical doctors who qualified from the NRMSM are progressing towards meeting community needs, there remains room for improvement. Overall, 29% (26/89) of NRMSM graduates have a mandated obligation to work at a district hospital because of community service, but only 27% (24/89) indicated a willingness to stay at a District Hospital (DH). This would suggest that training at NRMSM may be effective in meeting short-term staffing needs, but this is not a long-term strategy if graduates are unwilling to stay beyond their obligated term. To provide insight into this dynamic, we compared graduates using select social accountability indicators from the THEnet framework, including medical doctors who are demographically similar to the population served, who remain working with assigned communities 5 years after graduation, and who pursue further specialisation and take on leadership roles.^23^ Our findings on the four social accountability indicators and retention of NRMSM graduates in DH were mixed when compared to graduates from other schools.

The Nelson R. Mandela School of Medicine mirrors the population served in terms of age and gender, but not ethnicity. It is a measure of social accountability to have a workforce that is representative of the population served.^31^ Research has found that doctors of a minority status are more likely to work in medically underserved areas.^32^ In this study, the medical doctor demographic characteristics mirror the population of the KZN province in terms of gender but not ethnicity (see Table 2).^30^ There appears to be an over-representation of white, Indian and mixed race graduates from all schools when compared to the province demographics. As Van Rensburg found in his research, ‘the majority of black Africans (75.5% or 25.2 million) rely on the public health sector, representing about 74% of the total population’.^14^ For graduates of NRMSM, there are no clear differences with regard to age or sex; however, more than two-thirds (65%) of black people indicated a desire to remain working at the district hospital. Other demographic characteristics, such as rural background and upbringing, are known to impact the decisions to work in a rural area.^10^ This is an area of future exploration for research on recruitment and retention.

Nelson R. Mandela School of Medicine graduates are the least likely to stay longer term. Only 14% of the 231 medical doctors surveyed were working at district hospitals 5 years after graduation. Graduates from NRMSM are less likely to stay beyond 5 years than graduates from non-South African schools. Bursary obligations and compulsory community service after graduation were the most common reason for working at a district hospital and may be effective human resource recruitment strategies, but they are mostly short-term methods. While prior research has shown that financial incentives impact decisions for rural placement,^26^ they have not been found to be the most influential motive for taking the placement and remaining in the community.^10,22^

While many NRMSM graduates stated that they planned to leave the DH to pursue a specialisation, the extent to which they do so is unclear. Intention to stay after the obligation was fulfilled did not differ for NRMSM graduates in comparison to other graduates. When we looked at the question, ‘do you plan on leaving your post?’, 40% of NRMSM students responded affirmatively, with 44% indicating that they were leaving to pursue an additional specialisation. The THEnet considers further specialisation to be an indicator of social accountability even if medical doctors ultimately go into private practice if it is pursued in order to address the needs of community and health workforce. However, there is currently no method at the NRMSM or other medical schools in South Africa for tracking what graduates do when they leave. Motivation for pursuing a specialisation and where they practice post-specialisation is an area of further study. Prior research has shown that a desire for specialisation impacts one’s intention to work in rural areas. Specifically medical doctors report that rural assignments come with a lack of access to continuing education and professional development opportunities,^10,22,33^ as well as the absence of support by specialist consultants.^22^

Nelson R. Mandela School of Medicine graduates and those from other South African schools were less likely to be in leadership positions at the DH compared to those from non-South African schools. The THEnet considers the number of graduates in healthcare leadership roles as an important indicator of social accountability because they are in a position to influence the health system reform.^23^ Graduates from non-South African schools who have been working for more than 5 years most often report being in leadership positions. Those planning to leave within the next 5 years are in lower positions of authority, which corresponds to new graduates with fewer years of experience. In response, NRMSM could implement leadership training within the medical school curriculum to differentiate between being a manager and being a leader, to empower students to advocate for change and to solidify interprofessional team skills. There is a growing body of literature on the importance of leadership training for medical students, which is an area of further research.^34,35,36^ Findings from ‘low resource’ countries would add a unique perspective to the evidence base. This research may help others to make a difference within their own schools by examining existing practice patterns to identify areas of improvement in training.

New graduates are difficult to retain in the public sector;^14^ therefore, the researchers looked at reasons for staying at the district hospital and whether medical doctors have been stationed there for more than or less than 5 years. Many studies have examined why doctors leave a community, but an understanding of motivations to work with the underserved, specifically in district hospitals, can guide health workforce policies to strengthen retention in areas of need. Obligations and incentives tie people to jobs in district hospitals (at least for a while), but more knowledge about the elements of work and lifestyle that effect retention in these areas is needed. In ‘Caring for a common future: medical schools’ social accountability’, Woollard specifically states that Africa deserves a companion piece, as this article is from the experience of North American and ‘wealthier’ schools.^37^ This review takes up this call by exploring how NRMSM is progressing towards social accountability through exploration of how medical doctors’ practice reality and future intentions. While a low percentage of graduates intend to stay, it is encouraging that reasons for staying beyond an obligation are enjoyment of the work and lifestyle. It is important to emphasise that no absolute or universal indicator exists to measure an ideal level of social accountability for all schools and in all contexts. However, there are frameworks to guide schools in taking proactive steps for reflective assessment on progress in anticipating and responding to community needs.

Rourke outlined four strategies that schools can undertake to meet societal obligations, one which is pertinent for this study: ‘Produce graduates with the appropriate knowledge, skills, and interest who will practice how and where needed in the medical school’s region/nation’.^38^ The THEnet’s social accountability mandates expand on these strategies by delineating specific aspirations and indicators for schools to both implement and measure.^23^ This assists in determining whether a socially accountable medical school curriculum impacts graduates’ practice intentions and their ability and commitment to carry these goals into their professional practice.^39^ Choosing the ‘right’ indicators depends on the medical school’s context and needs of the community. While staying on in underserved communities is one measure of social accountability, it is one of several measures. The goal is for graduates to have the competencies necessary to address priority health needs of *the communities* they serve.

Family medicine specialists are perfectly positioned to lead this change as they are trained to address not only the health needs of the individual, but also the health needs of the community. These specialists are expert generalists who are trained to care for the majority of health problems seen in both clinics and district hospitals. The NRMSM has a strong emphasis on community immersion through the FM, but the results of this study suggest the need for greater emphasis on the overall curriculum content. This requires embedding the curriculum with socially accountable values and shifting from traditional learning that focuses exclusively on the individual biomedical model of disease to one that promotes social accountability. A socially accountable health professional curriculum is geared towards transformational learning to produce graduates with competencies to address priority health needs in the communities they serve. In a comprehensive systematic review of US primary care practice location, multiple factors were identified as predictors of practice in rural or underserved areas.^40^ Factors cited as strong contributors to medical doctors practicing in areas of need include medical school curriculums and programmes that have a specific focus on *rural, underserved, primary care* and *family medicine.* Similar curriculum elements aimed at improving recruitment and retention of medical doctors to underserved areas, in particular rural areas, may be relevant to South Africa.^41^ If medical doctors are leaving to obtain additional skills and further training or do not think that they are equipped to work with the underserved, then the curriculum content and the context where it is delivered needs to be re-examined.

### Limitations of the study

The focus of this study was on district hospitals only, which did not capture whether graduates are working in other underserved facilities. Data represented most but not all district hospitals and just under half of eligible medical doctors (44%) responded. In addition, some respondents had answers that were difficult to interpret. For example, when asked ‘what is your reason for leaving your post?’, some stated ‘travel’, which could mean travel makes the workplace undesirable or that they want to spend time traveling rather than working. Some respondents cited ‘training’ as a reason for leaving, which could mean that they want to pursue additional training on their own or training in the work environment which is not sufficient. While understanding the specifics may not significantly change the results, they could add evidence for future policy, education and workplace recommendations. Another limitation is that the specific curricular details of the non-NRMSM schools represented in this study are unknown.

## Conclusion

An expected return on investment in medical education, from a societal standpoint, is future access to clinicians who are not only technically competent but also ‘fit-for-purpose’, that is, they are able to meet the needs of the communities in which they train, learn and serve. Although the annual intake of NRMSM students mirrors the ethnic and gender demography of the population in the KZN province, the medical doctor workforce in KZN district hospitals is not ethnically reflective of the population served. The healthcare system must recruit and retain young and ethnically diverse health professionals, not only for continuity of care but also for ensuring adequate access to care within the population served. It is encouraging that graduates desire to pursue further specialisation, but without a means to track what graduates do afterwards, we are not able to conclusively state this as a socially accountable indicator. To assist in evaluating progress towards social accountability, future research should determine whether graduates returning to the KZN province after achieving their specialisation choose to practice in other underserved areas. Other areas of future research will look at analysing specific components of NRMSM, including practice intentions of medical students at different points in their education. Findings from these studies will contribute to the development of South African, in particular, NRMSM’s, education programmes and the goals of achieving health equity through education, research and services responsive to community priorities.
